# CRISPR/Cas9: the Jedi against the dark empire of diseases

**DOI:** 10.1186/s12929-018-0425-5

**Published:** 2018-03-28

**Authors:** Sehrish Khan, Muhammad Shahid Mahmood, Sajjad ur Rahman, Hassan Zafar, Sultan Habibullah, Zulqarnain khan, Aftab Ahmad

**Affiliations:** 1 Institute of Microbiology University of Agriculture, Faisalabad, Pakistan; 2 Center of Agricultural Biochemistry and Biotechnology, University of Agriculture, Faisalabad, Pakistan; 3 Department of Biochemistry/U.S.-Pakistan Center for Advanced Studies in Agriculture and Food Security (USPCAS-AFS), University of Agriculture Faisalabad (UAF), Faisalabad, 38040 Pakistan

**Keywords:** CRISPR/Cas9, Disease modeling, Genetic diseases

## Abstract

Advances in Clustered Regularly Interspaced Short Palindromic Repeats/CRISPR associated system (CRISPR/Cas9) has dramatically reshaped our ability to edit genomes. The scientific community is using CRISPR/Cas9 for various biotechnological and medical purposes. One of its most important uses is developing potential therapeutic strategies against diseases. CRISPR/Cas9 based approaches have been increasingly applied to the treatment of human diseases like cancer, genetic, immunological and neurological disorders and viral diseases. These strategies using CRISPR/Cas9 are not only therapy oriented but can also be used for disease modeling as well, which in turn can lead to the improved understanding of mechanisms of various infectious and genetic diseases. In addition, CRISPR/Cas9 system can also be used as programmable antibiotics to kill the bacteria sequence specifically and therefore can bypass multidrug resistance. Furthermore, CRISPR/Cas9 based gene drive may also hold the potential to limit the spread of vector borne diseases. This bacterial and archaeal adaptive immune system might be a therapeutic answer to previous incurable diseases, of course rigorous testing is required to corroborate these claims. In this review, we provide an insight about the recent developments using CRISPR/Cas9 against various diseases with respect to disease modeling and treatment, and what future perspectives should be noted while using this technology.

## Background

The potential and versatility of the field of genome engineering are remarkable in the sense of how scientists can utilize it for numerous benefits of mankind. The field has a wide range of applications in therapeutic medicine and biomedical research. The most pivotal aspect of genome engineering is certainly gene therapy, which can provide novelty to the way infectious diseases and genetic disorders are treated. To provide a cure through gene therapy, it is fundamental to study the gene functions and gene regulations through disease models that are in vivo and ex vivo. Another aspect of gene therapy is the way the genome is modified using different approaches, and how this modification could result in either a cure or a harmful mutation.

The genome whether in eukaryotes, prokaryotes or archea is a fascinating plethora of genes with endless protein products and possibilities. The vastness of proteins encoded by genes can be comprehended by a paradigm that twenty thousand proteins can be encoded by genes accumulated in only a meter of linear DNA in the genome [1]. In addition, this DNA also contains non-coding genes too. So, an estimate of the vastness of genes in the genome is tangibly comprehensible. In genetics, data from various studies of the past decade has elaborated the importance of variants and disease. Scientists have apprehended about the pivotal role that genome editing could play in the cure or prevention of infectious diseases. In the field of genome engineering, the term CRISPR/Cas9 has gained much fame in the previous few years. Many research papers are being written and published regarding exceptional experimentation using the technique: also claims of how this innovative, but simple method will prove to be the therapeutic answer to previous incurablediseases. Much testing is being done to confirm the claim of being the divine cure to diseases; in this review, we highlight the advancements that have been made using CRISPR/Cas9 in relation to cancer, genetic diseases, neurological, immunological disorders and viral infections.

## Primordial genome editing to CRISPR/Cas9: The journey

The journey of genetic modification through the past few decades has been remarkable and fascinating. First and foremost, the classical experimentation of Capechhi must be reminisced. He was the first modifier of genes in mammalian cells through his revolutionary research termed, “heteroduplex induced mutagenesis” [2]. In concise, he made possible genetic modification in cells, which potentially paved the way for future genomic modification research. However, modification of the genomes has come a long way since the revolutionary discovery by Capecchi, Many improvements have been indoctrinated into methodologies that have uplifted the technology of genomic modification to a higher level. For many years the field of genetic engineering was based only on simple homologous recombination of DNA, and there was a seemingly limited application of the field due to the requisite of more complex targeting and construct selection.

The consequent development of homologous combination of DNA based upon phages (bacterial viruses) simplified the engineering of much larger DNA fragments, and also made possible the production of target vectors [3]. The headway towards more accomplished gene modification got better when it was demonstrated that double-stranded breaks (DSB) could be induced in mammalian chromosomes [4]. It was further proved that the use of the meganuclease, ‘I-SceI’ could induce double-stranded breaks increasing the probability to get targeted homologous recombination events [5]. These meganucleases can be cogitated as modified forms of naturally occurring restriction enzymes having extended DNA recognition sequences (14–40 bp) [6]. The engineering of meganucleases is an arduous challenge because the DNA recognition and cleavage function of these enzymes are interwined in a single domain [7].

Further facilitation of genome editing was provided by the use of zinc finger nucleases (ZFNs). These ZFNs have two independent regions: a recognition domain of zinc fingers which identify the target triplet nucleotides in the DNA, while the second region, which is a non-specific nuclease called FokI generates the double stranded breaks (DSB) Since the nuclease has to dimerize to remain active, the ZFNs have to be used in pairs [8]. The ZFNs are small like MNs, but the designing of the recognition domain of ZFNs is more straightforward than MNs. More studies indicated about the potentiality of the use of ZF domains as an effective nuclease system; a target sequence of about 9 bp or 18 bp can be modified using ZFNs in a precise manner [9, 10].

Another genome editing tool is Transcription activator-like effector nucleases (TALENs). These have two independent parts. The first part consists of transcription activator-like factors (TALEs); these proteins were first discovered in the plant pathogen bacteria *Xanthomonas* [11]. During the TALEs infection of plants, these TALEs are transported into the plant cells and bind to DNA sequences resulting in modulation of the expression of the plant genes [11, 12]. These TALEs can be fused to a FokI nuclease domain, which in turn can create DSB in the targeted DNA. The designing of TALENs is simpler than ZFNs, while longer recognition sites enhances its specificity and make it less prone to off-target mutations [13].

Another technology is RNA interference (RNAi), which has also been used to some extent for gene expression modification. But, this technique has certain limitations. The effects of RNAi are generally non-specific, temporary and the technique is restricted to the knocking down of only transcribed genes [14]. These chimeric nucleases ZFNs, TALENs, and meganucleases possess powerful attributes to perform site-specific genome modifications, activation/inactivation of genes, sequence deletion, andrearrangement of the chromosomes [15]. However, an even more efficient genome modification tool was soon to be put to use to modify genomes.

## CRISPR/Cas9 miraculous genetic tool

In 2012, the field of genome engineering had one of the most important discoveries ever. Surprisingly, it involved the adaptive immune system of a Gram-positive bacteria *Streptococcus pyogenes*. The adaptive prokaryotic immune system CRISPR/Cas is present in 90% of archea and around 50% of bacteria [16]. The immune system is somewhat analogous to mammalian systems in remembrance of the foreign DNA; a sort of record is kept of prior exposures to phages and plasmids. A recurrent exposure results in a rapid and robust immune response to the invading foreign DNA. The genetic locus of the CRISPR/Cas systems is called “CRISPR array”; the locus contains a base pair range of ~ 20–50 (bp) separated by variable short DNA sequences termed as “Spacers”. These spacers are preceded by a leader sequence rich in AT. The sequence of DNA in the invading microbe possesses a sequence identical to the spacers, this foreign sequence is termed as “Protospacer” [17].

The mechanism of immunity generally involves three important phases: adaptation, expression (biogenesis of crRNA) and interference [18]. The first phase, which involves the injection of foreign DNA into the host, the adaption system selects protospacers from the foreign DNA and includes them into the CRISPR locus (array) towards the leader end. During the expression (crRNA biogenesis) phase, there is transcription at the CRISPR locus normally as a single pre-crRNA, which subsequently proceeds into a mature crRNA containing a single spacer. The final phase is the interference in which the crRNA guides the Cas nucleases to preciselyidentify and cleave the foreign nucleic acid [19]. A comparison of CRISPR/Cas9, ZFNs, meganucleases, TALENs and RNAi is given in Table 1.

**Table 1:** Comparison of CRISPR/Cas9, ZFNs, TALENs, meganucleases and RNAi

	CRISPR/Cas9	ZFNs	TALENs	Meganucleases	RNAi
Target site	19–22 bp	18–36 bp	24–40 bp	14–40 bp	Target site should be located 50–100 nt from ATG
Retargeting possibility	Easily retargeted without any complexity	Yes, but requires complex molecular cloning	Yes, but requires protein engineering	Yes, by protein engineering	Yes
Nuclease	Cas9	FokI	FokI	I-SceI	Dicer and Argonaute proteins
Recognition mechanism	RNA-DNA	Protein-DNA	Protein-DNA	Protein-DNA	RNA
Targeting restrictions	Protospacer adjacent motif (PAM) must be present	Non-G-rich sequences are difficult to target	T in the start and A at the end	Novel sequences are difficult to target	Only targets mRNA
Efficiency	High	High	High	High	High
Limitations	Off targets	Both expensive and time consuming to construct	Takes long to construct	Limited versatility in targeting	Off targets
Cytotoxicity	Low	Low	Variable to high	Low	Variable to high
Multiplexing ease	High	Low	Low	Low	High
Cost	Low	High	Moderate	Low	Low

## Classification of the CRISPR/Cas system

**Fig. 1:**
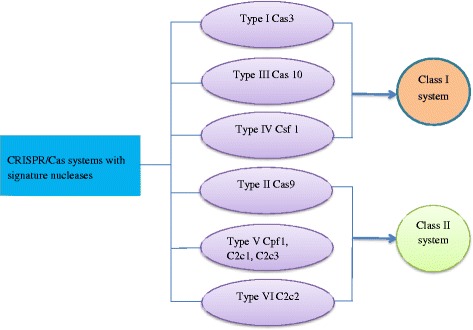
Various CRISPR/Cas systems have different signature endonucleases. CRISPR/Cas has six types and is divided into two classes. The class I system contains type I, III and IV, while the class II system comprises of type II, V, and VI. The CRISPR/Cas9 system is a type II of the class II system

The diversity of the CRISPR/Cas system is an essential component keeping in mind the wide range of foreign genetic elements that have to be confronted by it. Moreover, there are major differences in the repeated sequences of the CRISPR loci; it also applies to the Cas sequences and overall architecture of the Cas operon [18]. To overcome this ambiguity and to provide a clearer picture of the CRISPR/Cas system, it has been classified into six main types and two main classes shown in Fig 1. Type I-III is better understood, whereas types IV-VI have been identified recently. In the type I system the Cas-3 nuclease-helicase is involved, the type II system has the nuclease Cas9, while the type III systems possess the least understood Cas10. Type IV system possesses an uncharacterized protein Csf1. Type V systems contain either Cpf1, C2c1or C2c3, which are very much similar to Cas9. Type V1 contains a large protein C2c2. Class 1 system comprises of type I, III and IV and the class 2 system comprises of II, V and VI [19].

## CRISPR/Cas9 in genome editing

**Fig. 2:**
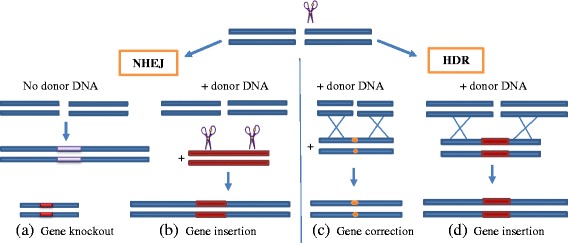
Comparison of NHEJ and HDR. The double-stranded breaks induced by nucleases can be joined by either homologous end joining or homologous directed repair. (**a**) The NHEJ mediated repair results in gene knockout without any donor DNA. (**b**) When donor DNA is available,it is cut by the nuclease simultaneously resulting in compatible overhangs; hence gene insertion may also take place by NHEJ. (**c**) HDR in the presence of donor DNA can be used for precise nucleotide substitutions resulting in modified genes. (**d**) HDR can also result in gene insertion

**Fig. 3:**
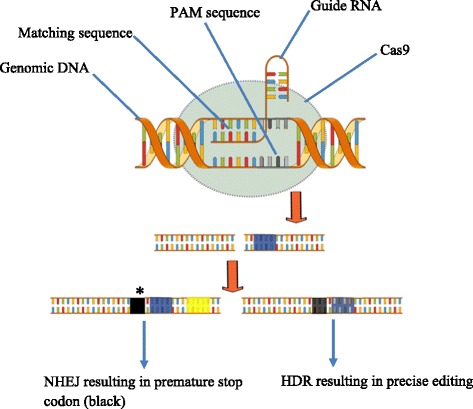
CRISPR/Cas9 mechanism. The important components in the system include Cas9 and gRNA. The nuclease Cas9 acts as a molecular scissors to cut the DNA strands. The gRNA directs the Cas9 to cleave the DNA at a specific position. The joining of the DNA occurs either by NHEJ or HDR

The potential of CRISPR/Cas9 was exploited in mammalian cells for the first time in 2013, the mechanism of action is similar to the prokaryotes with the single guide RNA (sgRNA) derived from the crRNA and trans-acting CRISPR RNA [20]. The CRISPR/Cas9 domains consist of sgRNA, and Cas9 nuclease that has RuyC and HNH as two catalytic active domains [21]. In response to Protospacer adjacent motif (PAM) present on the other strand, the sgRNA directs Cas9 through base pairing to the target site resulting in DSBs generated by Cas9. If homologous sequences are available these DSBs are repaired by homologous directed repair, the absence of homologous sequences will result in non-homologous end joining (NHEJ). The type of joining is pivotal as HDR results in an accurate gene correction while NHEJ may produce insertions/deletion mutations, shown in Figs. 2 and 3.

The reprogrammable property of Cas9 is incredible; it can be reprogrammed through inactivation of either or both HNH or RuvC into nickase Cas9 and dead Cas9 (dCas9). The dCas9 is catalytically inactive, but still shows a promising platform for targeting DNA through RNA guidance [22]. The CRISPR technology for gene regulation is termed as CRISPR interference (CRISPRi for gene repression) or CRISPR activation (CRISPRa for activation). Both use dCas9 fused with transcriptional repressors and activators [23]. In bacteria, dCas9 alone with sgRNA can efficiently silence gene expression [24]. However, only moderate silencing takes place in mammalian cells when dCas9 is used alone [24, 25] The fusing of dCas9 to the repressive KRAB (Krupel associated box) domain of Kox-1 exhibits strong gene silencing [25]. The effective targets sites of CRISPRi include proximal promoters, enhancers and coding region downstream from the transcription site of a gene [25]. The fusion of dCas9 with a transcription activator VP64 can result in the activation of a reporter gene [26, 27].

The CRISPR/Cas9 has many benefits in comparison to TALENs, ZFNs, RNAi and meganucleases. Firstly, in order to target a new DNA sequence the only requirement is a sgRNA, this is much simple and easy as compared to the synthesis of a cumbersome guiding protein for TALENs, ZFNs, RNAi and meganucleases. Furthermore, multiple sgRNAs can be used in the case of CRISPR/Cas9 to target different genomic loci simultaneously this is termed as “multiplexing” [28].

## Methods of delivery of CRISPR/Cas9

Both viral and non-viral delivery methods are being used for the delivery of CRISPR/Cas9 components into cell lines and animal models. Viral vectors such as self-inactivating lentivirus, adenovirus and adeno-associated virus (AAV) are potential delivery vehicles for CRISPR/Cas9. For non-viral delivery potential cargoes include plasmid DNA, Cas9/gRNA ribonuleoprotein complexes and donor nucleic acid templates [29]. However, for non-viral delivery various methods such as electroporation, induced osmocytosis, hydrodynamic delivery and lipid-mediated transfection can be used [30].

## CRISPR/Cas9 resources

Hundreds of online methods are available for CRISPR/Cas9 gene editing, construct designing with double stranded breaks, single stranded breaks, functional knockouts, plasmid with active gene expression, repress gene expression**,** tagging protein, finding target sequences and many others. A large number of resources have been developed which are being used for application in genome engineering, for identification of CRISPR target site and for selection of gRNA. Multiple online resources are available forcommercially available kits/ plasmids and CRISPR/Cas9 construction, as few are mentioned in Table 2.

**Table 2:** Commercial available kits/plasmids and services for CRISPR/Cas9 construction

Sr No.	Company Name	Web link
1	Addgene	www.addgene.org/crispr/guide/
2	Thermo Fisher Scientific	www.thermofisher.com/pk/en/home/life-science/genome-editing/geneart-crispr/crispr-libraries/lentiarray-crispr-libraries/lentiarray-cas9-lentivirus.html
3	ATUM	www.atum.bio/products/expression-vectors/mammalian?exp=5
4	Synthego	www.synthego.com/products/synthetic-sgrna/
5	GeneCopoeia	www.genecopoeia.com/product/transgenic-mouse/
6	Origene	http://www.origene.com/CRISPR-CAS9/
7	Clonetech	http://www.clontech.com/US/Products/Genome_Editing/CRISPR_Cas9/Resources/About_Guide-it_Kits
8	Sigma-Aldrich	https://www.sigmaaldrich.com/webapp/wcs/stores/servlet/LogonForm?storeId=11001
9	CHOPCHOP	https://chopchop.rc.fas.harvard.edu/
10	Active Motif	http://www.activemotif.com/catalog/1172/enchip

Numerous vectors are used for Cas9 according to the desired gene modification to be performed. The desired modifications include single strand break (SSB), double strand break (DSB), activation of gene expression, repression of gene expression and tagging of proteins knockout genes, these tools working so that any user can design construct with selectable marker, and different gene to be inserted according to their own demands. Many of them are freely accesible, but some are paid as well, depicted in Table 3.

**Table 3:** Selective CRISPR/Cas9 plasmids

	Name	Gene/insert	Selectable marker	Purpose	Reference
1.CRISPR/Cas9 plasmids that create single stranded break (Mammalian)	pX335-U6-chimeric_BB-CBh-hSpCas9n (D10A)	Humanized *S. pyogenes* Cas9(D10A) nickase	None	A human codon-optimized SpCas9 nickase and chimeric guide RNA expression plasmid	[28]
2.CRISPR/Cas9 plasmids that create double stranded break (Mammalian)	pX330-U6-chimeric_BB-CBh-hSpCas9	humanized *S. pyogenes* Cas9	None	A human codon-optimized SpCas9 and chimeric guide RNA expression plasmid	[28]
	pSpCas9 (BB)-2A–Puro (PX459) V2.0	hSpCas9-2A-Puro V2.0 (Synthetic)	Puromycin	Cas9 from *S. pyogenes* with 2A–Puro, and cloning backbone for sgRNA (V2.0)	[31]
3.CRISPR/Cas9 plasmids which activate gene expression(Mammalian)	SP-dCas9-VPR	SP-dCas9-VPR (*Homo sapiens*)	Neomycin (select with G418)	SP-dCas9 with VP64-p65-Rta (VPR) fused to its C-terminus; mammalian vector	[33]
	dCAS9-VP64_GFP	dCAS9(D10A, H840A)-VP64_2A_GFP (Synthetic)	GFP	Expresses dCas9-VP64 activator with 2A GFP	[34]
	lenti dCAS-VP64_Blast	dCAS9(D10A, N863A)-VP64_2A_Blast (Synthetic)	Blasticidin	3rd generation lenti vector encoding dCas9-VP64 with 2A Blast resistance marker (EF1a-NLS-dCas9(N863)-VP64-2A-Blast-WPRE)	[34]
4.CRISPR/Cas9 plasmids which repress gene expression(Mammalian)	pSLQ1658-dCas9-EGFP	dCas9 fuse to EGFP(*Homo sapiens*)	Puromycin	Template for NLS-dCas9-NLS-EGFP fusion protein for CRISPR imaging (the recipient vector can beTetON 3G promoter system)	[35]
	pLV hUbC-dCas9-T2A-GFP	Humanized dead Cas9T2A GFP (Other)	Zeocin	Co-expresses human optimized *S. pyogenes* dCas9 and GFP	[36]
	pJMP1	dCas9 (Other)	None	*Bacillus subtilis* dCas9 expression vector; integrates into lacA/ganA	[37]
5.CRISPR/Cas9 plasmid for tagging protein	Protein pFETch_NCoA2	Human	Donor plasmid pFETCh_NCoA2	Donor vector for 3’ FLAG tag of human NCoA2	[38]
	pFETch_RAD21	Human	pFETCh_RAD21	Homology arms and 3X Flag with P2A Neofor 3′ tagging of human RAD21	[38]

In addition, miscellaneous online resources are available for the designing of sgRNAs which provide information about OTs without limiting the PAM or number of mismatch bases, for finding potential off targets in any genome, identification and ranking all sgRNA targets sites according to off target quality, help in inquiry of guide sequences [32], and few of them are described in Table 4 with pros and corns.

**Table 4:** Tools available for sgRNA designing

Tool	Website	Purpose	Input	Output	Available genomes	Pros	Cons	Reference
Atum	https://www.atum.bio/eCommerce/cas9/input	Candidate gRNA	DNA sequence, (max 10,000 bp) gene name, genomic region	Candidate guidesequences and off target loci	5	Uses ATUM scoring to minimize off-targets/all in one nickase ninja	Not free of cost	N/A
Benchling	https://benchling.com/crispr	Candidate gRNA	DNA sequence/gene name	Candidate guidesequences and off target loci	5	Free of cost		N/A
CRISPR design	http://crispr.mit.edu/	Used to find target sequences for a sequence or seuqnces (batch mode), also provides in depth on/off target information	DNA sequence/FASTA files single or batch mode	Candidate guide sequences and off target loci	16	Helpful to generate many candidates with on/off information	Only handles short sequences up to23–500 bp/slow to use/no efficacy merit/does not indicate identity of mismatches	[39]
CHOPCHOP	https://chopchop.rc.fas.harvard.edu/	To find target sequences for a single sequence/gene/transcript	DNA sequence/gene name/genome location	Candidate guide sequences and off target loci,	23	Good to generate multiple guides for a single target, free of cost	No on-target efficacy	[40]
Cas off finder	http://www.rgenome.net/cas-offinder/	Provides information about OTs without limiting the PAM or number of mismatch bases	sgRNA	Off target loci for guide sequences	20	Free of cost/easy to use	Does not give indication if OTs are in coding sequences	[41]
Fly CRISPR	http://tools.flycrispr.molbio.wisc.edu/targetFinder/	Candidiate gRNA, software provides maximum stringency-(uses strict algorithm based on OT cleavage in cells lines) and minimum stringency; off target cleavage effects observed	DNA sequence	Candidate guidesequences and off target loci,	18	Free of cost	Slow to use	[42]
E-CRISP	http://www.e-crisp.org/E-CRISP/designcrispr.html	Finds target sequences for a single gene or sequence	DNA sequence/gene symbol	Candidate guide sequences and off target loci,	30	Free of cost	Numerous options maybe confusing/no account for identity of mismatches	[43]
Cas OT	http://eendb.zfgenetics.org/casot/	To find potential off targets in any genome	FASTA files	sgRNA and OT sites	User input	Free of cost/ First tool that identifies off-targetes in a user specified genome	No account for identitiy of mismatches	N/A
CRISPR ERA	http://crispr-era.stanford.edu/	Used for genome wide screening based on CRISPR, CRISPRi and CRISPRa	DNA sequence, gene name or TSS location	Candidate guide sequences and distances to TSS	9	Free of cost/can also be used for genome imaging and CRISPR synthetic circuit design		[44]
CCTop	http://crispr.cos.uni-heidelberg.de/	It helps in identifying and ranking all sgRNA targets sites according to off target quality	DNA sequence/FASTA file single/batch/ sequences 23 to 500 bp	Scores OTs and also ranks sgRNA by OTs	45	Free of cost/easy to use	No on–target efficacy prediction	[45]
WU-CRISPR	http://crispr.wustl.edu/	Potential candidate gRNA	DNA sequence/gene symbol	sgRNA list ranked by efficacy score	2 (Human and mouse)	Free of cost/easy to use	No account of identity of mismatches	[46]
GTscan	http://gt-scan.csiro.au/	Used to find target sequences and OTs for a single sequence	DNA sequence/FASTA file	SgRNA, genomic sites with 0 to 3 mismatches	51	Free of cost/easy to use	Trouble in finding exact matches in genome	[47]
CRISPR direct	https://crispr.dbcls.jp/	To find target sequences for a single transcript/sequence	DNA sequence/genome location/transcript	Target sequence and position	20	Free of cost/rapid visual display of target sequence and OT information	No on-target efficacy	[48]
COD	http://cas9.wicp.net/	Used to find target sequences for an input sequence	DNA sequence up to 400 bp	Gene bank file/CSC file/ OT scoring	27	Free of cost/easy to use/ OT scoring	Slow to use/no on-taget prediction	N/A
sgRNA scorer 2.0	https://crispr.med.harvard.edu/	Used to find target sequences/OT using Casfinder	DNA sequence/FASTA files upto10 kb	Target sequence with activity score	14	Free of cost/ allows to identify target sites for any CRISPR system	Slow to use/OT prediction does not account identity	N/A
CRISPOR	http://crispor.tefor.net/	To find candidate guide sequences	DNA sequence 1000 bp	Guide sequence with specificity score/guides for OTs	146	Free of cost	N/A	[49]

## CRISPR/Cas9 and disease resistance

### Development of cancer models using CRISPR/Cas9

A correct cancer disease model is highly essential to study and understand cancer pathogenesis. The same complex genetic scenario, as in cancer has to be restructured in various models of animals and human cells. For this purpose, CRISPR/Cas9 has proved to be an extremely valuable genetic tool for creating a same cancer-like conditions. The efficient CRISPR tool has expedited gene modification for the development of quick animal and human cellular models for oncogenic studies [50, 51]. Genome alterations are the driving force behind the processes that initiate human cancer. These cancer-initiating processes include chromosomal arrangements (deletions, duplications, inversions, translocations) and point mutations, which in turn inactivate tumor suppressor genes (TSGs) and convert proto-oncogenes into oncogenes [52]. The CRISPR/Cas9 system has been successfully used in established cell lines, organoids and patient-derived xenografts to engineer LOF(loss of function) mutations by NHEJ, GOF(gain of function) mutations by HDR and chromosomal re-arrangements by cutting at two distant loci [50, 52]. Several groups have used the CRISPR system to study hematological malignancies by CRISPR/Cas9 mediated editing of genes in hematopoietic cells and subsequent transplantation back into animals to assess tumorigenicity [53].

CRISPR/Cas9 has the potential to generate quick and efficient mouse models for cancer gene studies. Positive results were reported about the generation of pancreatic cancer in adult mice using a transfection-based multiplex delivery of CRISPR/Cas9 components. This allowed multiple genes in the individual cells to be edited. In addition, the authors also claimed to have modeled complex chromosomal arrangements, and a LOF mutation [54].

Mammalian hematopoietic stem cells (HSCs) have both multipotency and self-renewal abilities. The first term relates to the ability of these cells to give rise to a collection of blood cells, while the latter term indicates their ability to give rise to other HSCs without differentiation [55]. Mutations in these stem cells give rise to cancer. A research group modified five genes in a single mouse HSC. This was performed by the delivery of combinations of sgRNAs and Cas9 with a lentiviral vector. The modification of the genes resulted in clonal outgrowth and myeloid malignancy in the mice similar to the human disease [56].

KRAS is an important oncogene present in about 30% of human cancers [57]. It is the most common mutated oncogene in non-small cell lung cancer (NSCLC) in humans. A lung cancer model based on a mutation in the oncogene Kras was achieved by a research group. The genome of tumor suppressor genes was edited using CRISPR/Cas9 resulting in LOF of the TSGs. This editing resulted in the loss of function of the tumor suppressor genes similar to the human oncogenic condition [58]. The work on kras by scientists is the initiative step towards treatment of human disease by involving diverse genome engineering.

The Cre-loxP technology has been used by researchers to generate cancer models in mice. Using CRISPR/Cas9 a research group induced tumor formation in mice 3T3 cells similar to the Cre-loxP system. Mutations (Indels) were induced in two cancer suppressor genes: Pten and p53 [59]. Using CRISPR/Cas9 technology many human cellular models have been constructed for detailed cancer pathogenesis studies. A detailed description of recent research about the role of CRISPR/Cas9 in cancer disease modeling is given in Table 5.

**Table 5:** Role of CRISPR/Cas9 in cancer modeling

Type of cancer	Method of CRISPR/Cas9 delivery	Conclusion	Reference
Pancreatic cancer	Transfection based multiplexed delivery into mice	Editing of multiple gene sets in pancreatic cells of mice	[54]
Acute myeloid cancer (AML)	Lentiviral based delivery into Hematopoirtic stem cells	Loss of function in nine targeted genes analogous to AML	[56]
Liver cancer	Hydrodynamic injection into wild type mice	Mutation in the Pten and p53 genes leading to liver cancer in mice	[59]
Breast cancer	Plasmid transfection into JygMC (mouse cell line)	The stem cell marker Cripto-1 was shown to be as a breast target	[60]
Pancreatic cancer	Lentivirus/Adenovirus based delivery intosomatic pancreatic cells of mice	Knockout of gene Lkb1	[61]
Lung cancer	Plasmid transfection into human cell line (HEK 293)	Chromosomal rearrangement among EML4 and ALK genes	[62]
Lung cancer	Lentivirus/Adenovirus mediated	Gain of function of KRS and loss of function of p53 and Lkb1	[63]
Colon cancer	Plasmid transfection into DLD1 and HCT116 cell lines (human)	Loss of function in protein kinase c subgroups	[64]
Colorectal cancer	Electropolation into organoids intestinal epithelium (human)	Loss of function and directed mutation in APC, SMAD4, TP 53 and KRAS genes	[65]
Gliobastoma Medulloblastoma	Postnatal PEI-mediated transfection and in utero electroporation into mice	Deletion of TSGs (Ptch1, Trp 53, Pten and Nf1)	[66]
Renal cancer	Renca (mouse cell line)	Knockout of TSG VHL to induce cancer	[67]

### CRISPR/Cas9 in direct cancer gene therapy

Previous research has suggested the potential of CRISPR/Cas9 in the treatment of cancer. The ability of cancer cells to develop resistance to chemotherapy drugs is a primary cause of failure of chemotherapy. The application of the CRISPR/Cas9 system to inactivate drug resistance genes in a given cancer is a potential therapeutic strategy to increase the efficacy of chemotherapy.

For instance, Tang and Shrager suggested an approach using CRISPR-mediated genome editing in the treatment of epidermal growth factor receptor (EGFR)-mutant lung cancer. They proposed a sort of personalized molecular surgical therapy molecular. In the proposed technique, the CRISPR/Cas9 system comprising of Cas9 and sgRNA expression plasmid, and donor DNA plasmid will be packaged into viruses and delivered to patients. Intravascular delivery of CRISPR/Cas9 has been suggested by the authors for metastatic lung cancer and intratracheally for localized lung cancer [68].

For cancer, until now, the role of CRISPR/Cas9 has been predominantly about the generation of cancer models in animals and cell lines. These cancer models are and will be highly advantageous in understanding oncogenic pathways, new markers of cancer progression, identifying novel tumor suppressor genes, and will definitely provide an improved and efficient repertoire of strategies for cancer therapies. For instance, transcriptomic studies using CRISPR/Cas9 revealed a novel TSG “FOXA2” in pancreatic cancer, which was previously not known to function as a TSG [69].

Radiotherapy has also been used in the treatment of cancer for a while. However, poor radiation sensitivity has been reported in tumors having mutations in the p53 and p21 genes. Correction of these mutations in the cancer cells and interruption of the cellular radiation injury repair pathway may be a potential alternative way to augment radio-sensitivity. A combination of radiotherapy and CRISPR/Cas9-mediated gene therapy with synergistic anticancer effects may become a promising therapeutic strategy for cancer therapy [70]. Another aspect of CRISPR/Cas9 in cancer therapy is to enhance the host cells immune response to cancer. This could be possible through CRISPR/Cas9 mediated modification of T-cells. The re-infusion of genetically modified T-cells into cancer patients has shown promising results in clinical trials [71] and could be a way forward for anti-cancer therapies.

Another potential way to used CRISPR/Cas9 in cancer therapy could be the development of genetically engineered oncolytic viruses (OVs). These OVs have anti-tumor properties and can kill the cancer cells without causing any harm to the normal cells [72]. The killing of the cancer cells takes place via virus-mediated cytotoxicity or by an increased anticancer immune response. CRISPR/Cas9 can play an important role in oncolytic viral therapy by addition of cancer-specific promoter to genes that are indispensable for viral replication, and inducing mutations in viral genomes [73]. In both pre-clinical models and clinical trials promising results have been reported about the use of OVs in cancer therapy [72].

Recently, a research group in China headed by Lu You at Sichuan University has held clinical trials using CRISPR/Cas9 in a patient suffering from lung cancer. In this clinical trial, immune cells from the patient were removed and the Programmed death (PD-1) gene, which encodes for the protein PD-1 was disabled. This protein PD-1 is used by the cancerous cells to keep the host immune response in check. This is the first report of human trials using the CRISPR/Cas9 in clinical trials on human patients [126].

### Genetic disorders and CRISPR/Cas9

The modification of germline is a conventional approach for the study of genome modification studies in animal models. Various researches in the past years have confirmed the efficiency of CRISPR/Cas9 as a probable method to overcome genetic diseases in humans via experimentation in animal and human cellular models. Targeted mutation using CRISPR/Cas9 can manipulate genetic material by deleting and replacing causal mutations, host mutations can also be induced that will provide protection to the host [74]. Regarding the various genetic diseases, CRISPR/Cas9 technology can be used with ease to treat monogenic diseases; where a correction in the culprit gene could reverse the genetic disease. On the other hand, polygenic diseases are not so straightforward, having multiple mutations in the genome; they possess a far strenuous challenge to treat in comparison to monogenic diseases.

Duchene muscular dystrophy (DMD) is an X-linked recessive disorder, and is caused by mutations in the dystrophin gene [75]. An mdx (point mutation in dystrophin gene) mouse model of Duchene muscular dystrophy was used in an experiment. The CRISPR editing in the germline resulted in the correction of the dystrophin gene mutation in the mosaic offsprings. The offsprings carried 2–100% of the corrected gene. Surprisingly; the extent of phenotype rescue surpassed the percentage of gene correction [76]. CRISPR/Cas9 has also been used to correct another genetic disease cataract in a mouse germline. The cataract phenotype is caused by a frame-shift mutation of one base pair deletion in exon 3 of Crygc (crystalline gamma c) [77].

Beta thalassemia is one of the most common genetic diseases in the world. Mutations in the human hemoglobin beta gene (HBB) give rise to this genetic defect [78]. Induced pluripotent stem cells (iPSCs) from human beta thalassemia patients were edited by a research group with a CRISPR/Cas9 system combined with the transposon *piggyback.* This resulted in the efficient correction of the HBB mutations; in the corrected IPSCs no off-target effects were detected and the cells exhibited normal karyotypes indicative of full pluripotency [79].

Another genetic disease sickle cell anemia affects around 300,000 neonates globally per year [80]. The disease occurs as a result of mutations in the sixth codon of the beta-globin gene [81]. To check the gene editing ability of CRISPR/Cas9 an experiment was performed by Li et al. [81]. They developed a novel hybrid reprogramming viral vector, rCLAE-R6 (HDAd/EBV) using Adenovirus/Epstein bar virus. Highly efficient footprint iPSCs were obtained after viral vector transduction of keratinocytes. After delivery of CRISPR/Cas9 with adenovirus, nucleoporation was done using a 70-nucleotide single-stranded oligodeoxynucleotide (ssODN) correction template. Furthermore, genome sequencing of the corrected iPSCs confirmed no off-target modifications, and no changes in tumor suppressor genes [81].

Tyrosinemia is a genetic disease caused by a mutation in the FAH gene in humans. The mutation leads to abnormalities in the enzyme fumarylacetoacetate hydrolase functioning and the enzyme cannot break down the amino acid tyrosine [82]. CRISPR/Cas9 was used in an experiment to correct the FAH mutation in liver cells in a mouse model of the human genetic disease tyrosinemia. Tail vein hydrodynamic injection was used for the delivery of CRISPR/Cas9 and homologous donor template into adult mice. The adopted therapeutic method may be applicable to human therapeutics, as it does not comprise of any embryo manipulations [83]. A description about the use of CRISPR/Cas9 in the correction of genetic diseases is given in Table 6.

**Table 6:** Overview of gene correction of genetic diseases using CRISPR/Cas9

Genetic disease	Method of CRISPR/Cas9 delivery	Conclusion/outcome	Reference
Tyrosinemia	Tail vein hydrodynamic injection into adult mice	Correction of Fah gene mutation (1 nt substitution)	[83]
Hemophillia A	Transfection based delivery into iPSCs	Inversion based correction of the blood coagulation factor VIII (F8) gene	[84]
Hemophillia B	Tail vein hydrodynamic injection into Fah mice	Correction of mutation in F9 gene	[85]
Cataract	Injection into Oocyte of mouse	Correction in mutation of CRYGC gene (1 nt insertion)	[77]
Sickle cell anemia	Adenovirus based transduction into human IPSCs	Correction in sixth codon of beta globin gene	[81]
Beta Thalassemia	Transfection and piggyback removal in IPSCs from patients	HBB mutations corrected (1 nt substitution 4 nt insertion)	[79]
Cystic fibrosis	Transfection into intestinal stem cells from patients	Correction of CFTR gene mutation (3 substitution)	[50]

### Viral diseases and CRISPR/Cas9

The therapeutic challenge of viruses is captivating these obligate parasites rely on host metabolic machinery to replicate. It is a much arduous task to treat viruses as compare to bacteria due to their unique nature and machinery. Antiviral therapy targeting various viral proteins showed promising results, but anti-viral drug failure is becoming common, however, scientists have recently used the CRISPR/Cas9 phenomenon against a congregation of pathogenic viruses.

#### Herpesviruses

Herpesviruses include human simplex virus 1 (HSV-1), human cytomegalovirus and Epstein-barr virus. Human cytomegalovirus-1 causes cold sores and herpes simplex keratitis. Human cytomegalovirus causes conditions in immune-compromised people, while Epstein-barr virus causes Hodgkin’s disease and Burkitt's lymphoma [86]. The CRISPR/Cas9 system has been used against the EBV. Cells derived from a patient with Burkitt’s lymphoma with latent EBV infection (Raji cells) showed a marked reduction in proliferation and decline in viral load as well as restoration of the apoptosis pathway in the cells after treatment with CRISPR/Cas9 [87]. In another research, CRISPR/Cas9-mediated editing of EBV in human cells was done using two gRNAs to make a targeted deletion of 558 bp in the promoter region for the BART (Bam HI A rightward transcript), which codes for viral miRNA’s. This resulted in the loss of BART miRNA expression and activity indicating the feasibility of CRISPR/Cas9-mediated editing of the EBV genome. No off-target cleavage was found by deep sequencing [88]. It was the first genetic evidence that the BART promoter drives the expression of the BART transcript, and also a new and efficient method for targeted editing of EBV genome in human cells.

#### Human papillomaviruses (HPV)

Human papillomaviruses (HPV) cause warts in humans; in addition, they are also oncogenic in nature. The majority of the cancers are caused by HPV16 and HPV18 including cervical cancer in females. The viral proteins E6 and E7 are the major contributors towards the oncogenic properties of the viruses; these proteins are encoded by the oncogenes E6 and E7 [89]. Kennedy et al. used HPV 16 and HPV 18 integrated HELA and SiHa cervical cancer cell lines for their CRISPR-associated editing of the E6 and E7 genes of HPV. They were able to induce mutations in the E6 and E7 genes rendering them inactive and promoting the anti-tumor effect of p53 and Rbp. They employed a CRISPR/Cas9 system comprising of Cas9, E6 and E7 specific gRNAs [90]. Future research should pay emphasis on using CRISPR/Cas9 to not only inactivate potential cancer risk genes, but to also promote anti-tumor factors.

#### Hepatitis B virus

Hepatitis B virus is among the major viruses of health concern. It causes liver cirrhosis and hepatocellular carcinoma in humans [91]. Anti-viral therapy has a major disadvantage against the virus; due to the fact that the covalently closed circular DNA of the virus localizes in the nucleus of hepatocytes [92]. Promising results have been reported in using CRISPR/Cas9 against hepatitis B virus. [91].

Moreover, a research team designed eight gRNAs against HBV and showed that the CRISPR/Cas9 system significantly reduced the production of HBV core and HBsAg proteins in the Huh-7 hepatocyte-derived cellular carcinoma cells transfected with an HBV-expression vector. Further, this system could cleave intrahepatic HBV genome-containing plasmid and facilitate its clearance in vivo in a mouse model resulting in a reduction in serum HBsAg level [93].

For the simultaneous targeting of the three loci of the HBV genome, a multiplex all in one CRISPR/Cas9 nuclease and Cas9 nickase vector systems was used in an experiment. [94]. Transfection of the HBV expressing plasmid and vectors into HepG2 cell line was performed. Results indicated a reduction in the HBV replicative intermediates, and also a reduction in the surface and envelope antigens. DNA sequencing confirmed fragmentation of the viral genome and no off-target mutations were reported either. The all in one vector represent an adaptable methodology for simultaneous targeting of the three HBV domains and may be used for therapeutic purposes for HBV patients [94].

In another experiment, lentiviral transduction of Cas9 and HBV-specific gRNAs into human cell line HepAD was performed. Effective inhibition of the HBV DNA production was observed. Total HBV DNA levels were reduced by up to ~ 1000-fold while cccDNA levels were reduced by up to ~ 10-fold, and the majority of the residual viral DNA was mutationally inactivated [95].

In the most recent study of HBV and CRISPR, Zhen et al., targeted the HBsAg and HBx-encoding region of HBV. The experiment involved both cell culture and in vivo trials. The level of surface antigen was much reduced as indicated by ELISA. The HBV DNA levels and HBsAg expression in mouse liver were reduced as also shown by qPCR and immunohistochemistry respectively [96]. The encoding regions of the hepatitis virus must be the center of concentration for future research, in which case inactivation of these encoding regions will certainly decrease the catastrophic effects of the hepatitis viruses.

#### Human immunodeficiency virus (HIV)

One of the most researched viruses in history is the HIV; the causative agent of acquired immunodeficiency syndrome (AIDS) in humans. For the past 30 years, AIDS has remained a major health concern [97]. Until much information has been gained about the pathogenesis replication and clinical manifestations of the virus, however, a complete therapeutic strategy has not been achieved so far. Presently, it is estimated that about 37 million people are infected with HIV globally, and each year there is a substantial increase in a number of the infected. In the past decade, AIDS related mortalities have been reduced due to the use of anti-retroviral therapy (ART) [98]. But, still, a proper cure of the virus has been unattainable.

There are two possible mechanisms of the inactivation of HIV gene expression using CRISPR/Cas9: 1. prior to virus integration into the host genome, Cas/9 can inactivate viral gene expression 2. Cas9 can cause disruption of the proviral element already integrated into the host genome. In general targeting of the long terminal repeats (LTR) of the virus has resulted in better results. The cause may be the presence of the conserved trans-activation response (TAR) sequence among HIV-1 subtypes, hence LTR should be the preferred targeting of future anti-viral strategies using CRISPR/Cas9 [99].

CRISPR/Cas9 has been used in research for HIV treatment with mixed outcomes. Wang et al., used CRISPR/Cas9 against HIV proviral infection in cells to initiate sequence-specific cleavage. Replication of the HIV was inhibited by harnessing the T-cells with Cas9 and anti-viral guide RNA’s, but the virus seemed to escape the inhibition. Sequencing results of the escaped HIV showed various nucleotide substitutions, deletions and insertions around the cleavage site indicative of NHEJ associated DNA repair, thus to some extent there is a limitation of the use of CRISPR/Cas9 against HIV [100].

Another interesting prospect in the battle against HIV is editing host cell factors that are deemed necessary for the HIV replication and infection in the T-cells. Examples of such host cell factors include CXCR4 (Chemokine receptor type 4) and CCR5 (Chemokine receptor type 5). For efficient entry of the virus into the cell, the envelope (Env) has to bind with these two receptors [101, 102]. Other factors are TNPO3 (transportin 3), required for viral replication, and LEDGF (lens epithelium derived growth factor), required for integration of the viral genome into host cells. In an experiment, electroporation of CRISPR/Cas9 ribonucleoproteins (RNPs) into primary CD4+ T cells resulted in CXCR4 or CCR5 knockout cells. These cells exhibited resistance to HIV infection in a tropism dependent manner. The knockout of LEDGF or TNPO3 resulted in reduced infection, but impartial to any tropism. CRISPR/Cas9 ribonucleoproteins can furthermore, edit multiple genes simultaneously enabling studies of interactions among multiple hosts and viral factors [103].

In further research on the effectiveness of CRISPR/Cas9 on HIV, a research included the targeting of the LTR, Gag and Pol gene. An HIV-susceptible human T-cell line was used, and transduction of the gRNA and Cas9 was done. A clear inhibition was observed in the early HIV infection. However, the anti-viral potency was insufficient in multiple rounds of the wild type viral replication, indicating difficulties in treating HIV with CRISPR/Cas9 [104].

In a transgenic mouse model, Kaminski et al. used an adeno associated virus 9 vector (rAAV_9_) expressing gRNAs and Cas9 for removing important segments of the HIV (5′ LTR and Gag gene). Tail vein injection in the mice exhibited cleavage of viral DNA and excision of a 978 bp DNA segment between LTR and Gag in various organs such as kidney liver, lung heart and also in blood lymphocytes. Retro-orbital inoculation excising of CRISPR/Cas9 resulted in of targeted DNA segment and also inhibited gene expression of the virus hence indicating for the first time, the in-vivo efficacy of CRISPR/Cas9 via rAAV_9_ in a wide variety of cells and tissues that harbored copies of the HIV DNA [105].

Table 7 gives an insight into the use of CRISPR/Cas9 against viral diseases.

**Table 7:** Overview of CRISPR/Cas9 in virus genome modification

Virus	Method of delivery of CRISPR/Cas9	Conclusion/outcome	Reference
HSV-1	Transfection into HEK293 cells	Modification of ICP0 gene in different locations of genome	[106]
EBV	Nucleofaction into Burkhitt’s lymphoma cell line	Complete virus clearance in 25% cells, partial in 50%	[87]
EBV	Transfection into HEK 293-BX1 and C666–1 cells	Loss of BART Micro RNA expression	[88]
HPV	Lentiviral transduction into HELA and SiHA cell lines	Indel mutations in the E6 and E7 genes	[90]
HBV	Transfection in to Huh cells	Cleavage of the HBV genome-expressing template	[93]
HBV	Hydrodynamic injection into C57BL/6 mice	Cleavage of the HBV genome-expressing template	[107]
HBV	Transfection into HepG2 cell line	Fragmentation of viral genome	[94]
HBV	Lentiviral transduction into HepAD cell line(Chronic HBV infection)	Inhibition of viral DNA production	[95]
HIV	Lentiviral transduction into SupT1 CD4+ T cell line	Inactivation of virus and acceleration of virus escape	[100]
HIV	Lentiviral transduction into T-cells	Inhibition of early phase viral infection, but anti-HIV potency was not consistent in multiple rounds	[104]
HIV	Retro-orbital injection into transgenic mice	Decrease of viral gene expression in T-cells	[105]
Polyomavirus (JCV)	Transfection into TC 620 cell line	Inactivation of T-antigen gene	[108]

#### Future of CRISPR/Cas9 against viral diseases

The original antiviral role of CRISPR/Cas9 in prokaryotes makes it an interesting candidate to use against human viruses. Many of the advancements regarding its role in antiviral therapy have already been discussed. For the development of antiviral therapies, CRISPR/Cas9 can be used to target the virus sequence for destruction or can be employed for the engineering of host sequences essential for virus infection [74]. Furthermore, CRISPR/Cas9 can be used to knockout host factors that may be essential for virus survival, integration and replication [103]. In addition to much comprehensive research on antiviral therapy, another dimension is the use of CRISPR/Cas9 in the development of vaccines for viral diseases. CRISPR/Cas9 system into vaccine development has been reported by Liang et al., who combined both CRISPR/Cas9 and Cre/Lox system for the development of a pseudorabies vaccine for swines [109]. Over passage expance of time the perspective of vaccine manufacturing using CRISPR/Cas9 is certainly a point to ponder for further research.

### Neurological disorders

These disorders are a menace to public health affecting millions of people worldwide. Potential treatment of neurological disorders may be futile, due to the chronic nature of the disorder and treatment ineffectiveness. Research is now being done on the potential role of CRISPR/Cas9 against neurological disorders. Huntington disease is a neurodegenerative disorder characterized by dementia, choreatic movements and behavior disturbances [110]. A novel CRISPR/Cas9-based gene editing approach was used against Huntington disease (HD) and resulted in the inactivation of HD-associated mutant HTT allele without affecting the normal allele [111].

Another neurological disorder Schizophrenia has also been tested upon using CRISPR/Cas9, using a mouse model a single intracranial injection of AAV2g9 vectors encoding guide RNAs targeting the schizophrenia risk gene MIR137 (encoding MIR137) was used. It resulted in brain-specific gene deletion with no detectable events in the liver. This engineered AAV vector is a promising platform for treating neurological disorders through gene therapy, gene silencing or editing modalities [112].

The use of CRISPR/Cas9 against neurological disorders has immense potential to be explored by scientists. However, some limitations have to be addressed while using the system in neurological disorders. First of all, efficient delivery of the Cas9 nuclease and sgRNA to the brain is essential, and novel methods have to be introduced that can lead to efficient gene insertion and correction in the post-mitotic cells of the brain. In addition to devising therapeutic strategies, this genome editor can certainly be applied in attaining a comprehensive notion about the working and functionality of the brain, and to get a more lucid understanding of the mechanisms of neurological disorders [113].

### Allergy and immunological diseases

CRISPR/Cas9 possesses potential against allergic and Mendelian disorders of the immune system. Janus Kinase 3 (JAK 3) deficiency in humans is characterized by normal but poor functioning B-lymphocytes, and the absence of natural killer cells (NKs) and T-lymphocytes. For correction of this immunological disorder, CRISPR/Cas9 was used in induced pluripotent stem cells. Correction of the JAK 3 mutation was made, resulting in restoration of normal T-lymphocyte development and number [114].

X-linked hyper immunoglobulin IgM syndrome is an immunological disorder of humans. It is caused by a mutation in the CD40 ligand and causes increased level of IgM. Kuo et al. have reported the correction of the mutation using CRISPR/Cas9 [115]. Another immunological disorder is X-linked chronic granulomatous disease (X-CGD) result due to mutation in the CYBB gene, this leads to improper functioning of the phagocytes. The NADPH oxidase system of the phagocytes of the patient is defective in this condition; as a result the phagocytes are unable to generate superoxide rendering them ineffective to kill pathogenic microbes [116]. Recently, CRISPR/Cas9 was used by a team of scientist who were successful in correcting the mutation in the CYBB gene of HPSCs from patients suffering from X-CGD [117].

Scientists are using methodologies, which provide a critical analysis of the use of CRISPR/Cas9 as a treatment for allergic and immunological diseases. Single nucleotide polymorphisms (SNPs) are known to contribute to allergic diseases such as asthma and allergic rhinitis [118, 119]. These SNPs can be modified using CRISPR/Cas9, however, much testing in experimental systems is necessary before advancing to human therapy. Additionally, hematopoietic cells remain the most common target for both allergic and immunological diseases, and can be corrected using CRISPR/Cas9 [115]. The main emphasis of CRISPR/Cas9 in relation to allergic diseases has been about the investigation of the potential role of particular genes. Using the technology, certain gene knockout models can be created, which will provide an evaluation of the role of certain genes in allergic diseases and immunological disorders. Moreover, CRISPR/Cas9 is rapidly becoming the primary tool to create mutant mouse models of diseases, including allergic and immunologic diseases, due to the ease, precision, and flexibility of this technique.

### Potential of CRISPR/Cas9 as antimicrobials

A diverse manner of using CRISPR/Cas9 could be putting it to use as an antimicrobial entity. Antibiotics have been used in the treatment of bacterial diseases for quite a while. They inhibit certain bacterial metabolic pathways and hence kill the microbe in different ways, but cannot target specific members of a microbial population. However, antibiotic resistance has been a major problem, and now the emergence of multidrug-resistant (MDR) bacteria is a ginormous menace.

Using CRISPR/Cas9 as an antimicrobial tool, Bikard et al. reported promising results that used a phagemid-based delivery of programmable, sequence-specific antimicrobials using the RNA-guided nuclease Cas9. The reprogrammed Cas9 only targeted the virulence genes of *Staphylococcus aureus* killing virulent strains, and did not kill avirulent strains. Much of the antibiotic resistance is caused by plasmids; the nuclease was also reprogrammed to target plasmid sequences in *S. aureus* with positive results. In a mouse skin model the CRISPR/Cas9 antimicrobials showed extreme potential in killing of *Staph aureus*. This technology creates opportunities to manipulate complex bacterial populations in a sequence-specific manner [120].

The true capability of CRISPR/Cas9 as an antimicrobial can be further exploited by developing delivery systems using phages that can help in the injection of cargo into diverse bacterial strains. However, broad host range phages are very rare and those that are known infect only single species within a genus. In molecular biology, phages have been serving as the first model system, but little is known in how to alter or expand the host range of the phages. This provides an excellent opportunity to develop enhanced phages that will have the ability to infect any host microbe. Alternatively, nanoparticles, or outer membrane vesicles may be used as delivery systems.

## Gene drive and CRISPR the ultimate gene editing alliance?

A gene drive is a process by which an altered gene is introduced inside an animal population. The aim of gene drive is to get desired traits a population through natural reproduction alone. The use of novel gene drives resides in the use of CRISPR, the CRISPR technique has great potential in genome engineering. By using it scientists edit genes with precision, quickness, and economy, in addition it also has the potential of generating genetic alterations in wild animals that may persist in nature [127].

Gene drive research and its applications are progressing quickly. The CRISPR/Cas9 phenomenon became the holy grail of genome editing about 4–5 years ago, and the first reports of gene drive organisms (yeast, laboratory fruit flies and mosquitoes) were published in 2015 [128]. It will take some time for scientists to release genetically modified organisms (GMO’s) with a gene drive system into the wild, till that happens the US National Academy of Sciences, Engineering and Medicine has recently approved comprehensive research for the betterment of gene drive and has encouraged carefully controlled field trials in the near future [129].

Gene drives have the potential to limit the spread of various diseases, to support the agriculture sector by reversing pesticides and herbicides resistance in insects and plants.. Till now there is no claim about the successful testing of any gene drive in the wild but in laboratory organisms like fruitfly and mosquitos, scientists have converted almost entire populations to carry a favored trait. In laboratory tests, different groups have already used CRISP for editing genes of mosquito species, these blood thirsty insects harbor the parasite that causes malaria, and so the gene drive can be used to prevent female mosquitoes from producing fertile eggs [129].

So far, gene drives have been tested and evaluated only in laboratories, and the main emphasis of research has been on mosquitoes that transmit infectious diseases, as well as lab animals such as mice. The objectives are numerous however, some of the pivotal ones include control of the size of the population, or to suppress it completely, the last but not least is its use to combat against infectious diseases. Gene drives therefore have the potential to reduce the occurrence of, and possibly eradicate various infectious diseases by upsetting their transmission chains [130].

## Conclusions

Ever since CRISPR/Cas9 was introduced as the key aspect of genome engineering a plethora of advances have been made. Despite its easy adoption, the proper translation of this technology for clinical purposes has been cumbersome. The main emphasis on the utilization of this genome-editing tool has been to develop a control of the repair mechanisms in the targeted DNA. Despite recent advances in genome editing targeted in vivo gene integration has not been achieved specifically in non-dividing cells A recent development of much interest is homology-independent targeted integration (HITI) for CRISPR/Cas9, which allows robust knock-in in both dividing and non-dividing cells [28, 121–123].

Another limitation in the use of CRISPR/Cas9 has been off-target cleavage activity. However, experiments have proven that shortening the length of gRNA < 200 nucleotides can reduce off-target mutagenesis. [124]. A high fidelity variant Cas9 termed (Cas9-HFI) has been constructed with reduced off-targets. It was compared with wild type Cas9, it showed similar on target results and reduced off-targets [124]. Similar results were shown by Slaymaker et al. by the use of an “enhanced specificity” Cas9 [125]. In short, the use of altered Cas9 nucleases, which possess higher precision as compared to wild type Cas9 could be an appropriate tactic to curtail off-target cleavage.

In this review, many of the possibilities of CRISPR/Cas9 have been outlined in relation to not only understanding the various diseases but also devising ways of making efficient therapeutic cures using CRISPR/Cas9. What the future holds with CRSIPR/Cas9 is both fascinating and intriguing, however much further research is necessary to overcome the shortcomings at hand, to tackle any possible adverse effects on humans, and the ethical aspects of such experiments must not be overlooked.
